# Treatment options in myocarditis and inflammatory cardiomyopathy: Focus on i. v. immunoglobulins

**DOI:** 10.1007/s00059-018-4719-x

**Published:** 2018-06-15

**Authors:** B. Maisch, P. Alter

**Affiliations:** 1 Fachbereich Medizin, Philipps-Universität Marburg und Herz- und Gefäßzentrum (HGZ) Marburg, Feldbergstr. 45, 35043 Marburg, Germany; 2 Klinik für Innere Medizin-Pneumologie und Intensivmedizin, UKGM und Philipps-Universität Marburg, Marburg, Germany

**Keywords:** Carditis, Inflammation, Cardiomyopathies, Treatment, Intravenous immunoglobulins, Karditis, Inflammation, Kardiomyopathie, Therapie, Intravenöse Immunglobuline

## Abstract

For myocarditis and inflammatory cardiomyopathy, an etiologically driven treatment is today the best option beyond heart failure therapy. Prerequisites for this are noninvasive and invasive biomarkers including endomyocardial biopsy and polymerase chain reaction on cardiotropic agents. Imaging by Doppler echocardiography and cardiac magnetic resonance imaging as well as cardiac biomarkers such as C‑reactive protein, N‑terminal pro-B-type natriuretic peptide , and troponins can contribute to the clinical work-up of the syndrome but not toward elucidating the underlying cause or pathogenetic process. This review summarizes the phases and clinical features of myocarditis and gives an up-to-date short overview of the current treatment options starting with heart failure and antiarrhythmic therapy. Although inflammation in myocardial disease can resolve spontaneously, often specific treatment directed against the causative agent is required. For fulminant, acute, and chronic autoreactive myocarditis, immunosuppressive treatment has proven to be beneficial in the TIMIC and ESETCID trials; for viral cardiomyopathy and myocarditis, intravenous immunoglobulin IgG subtype and polyvalent intravenous immunoglobulins IgG, IgA, and IgM can frequently resolve inflammation. However, despite the elimination of inflammation, the eradication of parvovirus B19 and human herpesvirus-6 is still a challenge, for which ivIg treatment can become a future key player.

## Zusammenfassung

Bei Myokarditis und inflammatorischer Kardomyopathie ist heute neben der bewährten Behandlung der Herzinsuffizienz eine ätiologisch begründete Therapie die beste Option. Voraussetzungen dafür sind die Bestimmung nichtinvasiver und invasiver Biomarker inklusive Endomyokardbiopsie und Polymerasekettenreaktion (PCR) auf kardiotrope Substanzen. Bildgebende Verfahren wie Echodoppler- und kardiale MRT-Untersuchung sowie kardiale Biomarker wie CRP, NT-proBNP, Troponin können die Diagnostik in Bezug auf das Syndrom ergänzen, zielen aber nicht auf den verursachenden Erreger oder den zugrunde liegenden autoreaktiven Pathomechanismus. Neben den Phasen und dem klinischen Bild der Myokarditis werden kurz die aktuelle Herzinsuffizienz- und antiarrhythmische Therapie dargestellt. Die spontane Rückbildung der Entzündung ist zwar möglich, oft wird aber eine spezifische Therapie erforderlich. Bei fulminanter, akuter und chronischer autoreaktiver Myokarditis hat sich gemäß der TIMIC- und ESETCID-Studie eine immunsuppressive Behandlung als nützlich erwiesen. Bei viraler inflammatorischer Kardiomyopathie und Myokarditis sind i.v.-Immunglobuline (IgG bzw. IgGAM, also polyvalente IgG, IgA und IgM) in der Lage, die Entzündung fast immer, die virale Ursache (Parvovirus B19 und humanes Herpesvirus 6, HHV6) bei der überwiegenden Zahl der betroffenen Patienten zu eliminieren.

In 2012 we reviewed the treatment options in (peri)myocarditis and inflammatory cardiomyopathy in a special issue of this journal devoted to heart failure and cardiomyopathies [1]. Now, 5 years later, it is timely and appropriate to take stock of old and new data on this topic.

## Evolution of diagnoses

In 2013, experts of the European Society of Cardiology (ESC) working group on myocardial and pericardial diseases published a position statement on “The current state of knowledge on aetiology, diagnosis, management and therapy of myocarditis” [2]. Specifically named causes of myocarditis were either infective or immune-mediated or toxic [2, 3]. Table 1 sums up the long list of possible causative pathogens and compares them with the real-world data of the Marburg Myocarditis Registry (MMR) comprising records of 1098 biopsied patients with suspected inflammatory dilated cardiomyopathy and/or myocarditis [1, 4]. The comments add important clues on how the diagnosis was made in the MMR. Not mentioned but self-evident are a full clinical work-up of the patient including a detailed history, electrocardiogram (ECG) at rest and at exercise, imaging by Doppler echocardiography or cardiac magnetic resonance imaging (MRI), as well as a complete laboratory examination with C‑reactive protein (CRP) as a marker of inflammation and N‑terminal pro-B-type natriuretic peptide (NT-proBNP) and high-sensitivity (hs) troponin T or I as cardiac biomarkers of heart failure and necrosis, respectively. Of note, cardiac MRI is an important method for clarifying the presence of inflammation or fibrosis in addition to function and pericardial effusion, but it cannot substitute endomyocardial biopsy for establishing an etiologically based diagnosis [1–5]. For the diagnosis of viral vs. autoreactive (nonviral) myocarditis and for the diagnosis of eosinophilic or giant cell myocarditis, endomyocardial biopsy remains essential, while the biopsy work-up includes histology, immunohistology, and polymerase chain reaction (PCR) for RNA or DNA viruses [1–6].

**Table 1:** Causes of myocarditis and inflammatory cardiomyopathy in the MMR^a^

	Infectious agent	% pos. in MMR	Comments Diagnosis made via:
1. Infectious myocarditis			
Bacteria	*Chlamydia pneumoniae*	0.03	Serodiagnosis
	*Mycobacterium tuberculosis*	0.02	IGRA (Quantiferon) or microscopy from sputum, pericardial fluid, in Africa more frequent
	*Haemophilus influenzae*	0.002	Serodiagnosis
	Staphylococci	0.03	Blood culture, in sepsis or endocarditis
	Streptococci	0.02	In rheumatic fever, in cooperation with Chandigarh
Spirochete	Syphilis	0.001	Serodiagnosis
	*Borrelia burgdorferi*	0.7	ELISA and Western blot or PCR from EMB
Rickettsia	*Coxiella burnetiid*	0.005	Serodiagnosis, predominant pericarditis
Fungi	Candida	0.002	In immunocompromised patients, diagnosed by culture
Protozoa	*Plasmodium falciparum* (malaria)	0.002	Microscopy (thick blood film)
	*Toxoplasma gondii*	0.002	Serodiagnosis
Helminthic infections	–	0	None in MMR
Viruses (RNA subtype)			
Picornaviruses	Coxsackie A + B	0.019	All by PCR, epidemiologic shift in late 1990s, none since 2002
	Echo	0.005	PCR
	Hepatitis B and C	0.002	Serodiagnosis or PCR
Orthomyxoviruses	Influenza A or B	0.002	Serodiagnosis
	H1N1	0.001	Serodiagnosis
Paramyxoviruses	Mumps	0.001	Serodiagnosis
	Measles	0.002	Serodiagnosis
Toga‑/Rubivirus	Rubella	0.001	Serodiagnosis
Flavi‑/Arbovirus	Dengue	0.001	Serodiagnosis
Viruses (DNA subtype)			
Adenoviruses	A1, 2, 3, 5	0.011	PCR
Erythroviruses	Parvovirus B19 types 1–3	28	PCR
	Herpesviruses: human herpes 6 virus	0.03	PCR; sometimes together with PVB 19 virus
	Cytomegalovirus	0.02	PCR or ISH
	Epstein–Barr virus	0.012	PCR
	Varicella zoster	0.001	Serodiagnosis
	Retrovirus: HIV	0.005	PCR or by serodiagnosis
	Rhabdovirus	0.001	–
2. Noninfectious myocarditis	Autoreactive myocarditis	53	Exclusion of microbial agents
Systemic autoimmune diseases	Giant cell myocarditis	0.03	Histology
	Wegner’s granulomatosis	0.01	Histology
	Sarcoid heart disease	0.015	Histology
	Rheumatoid arthritis	0.03	Histology and serology
	Sjögren syndrome	0.02	Serology
	Systemic lupus	0.05	Serodiagnosis
	Crohn’s disease	0.02	Serodiagnosis
	Dermatomyositis	0.02	Serodiagnosis
	Kawasaki syndrome	0.015	–
Rejection	After heart transplantation	1	In cooperation with Hannover Medical School
	After stem cell transplantation	0.002	–
Hypereosinophilic syndrome (HES)	Löffler’s endomyocarditis	0.01	Biopsy and histology
	Churg–Strauss syndrome	0.01	Biopsy and histology
3. Toxicity			
Alcohol	Alcoholic cardiomyopathy	0.2	History, negative PCR on microorganisms
Drug toxicity	Aminophylline, amphetamine, anthracycline, chloramphenicol, cocaine, cyclophosphamide, d5-fluorouracil, mesylate, methyl sergide, phenytoin, trastuzumab, zidovudine, ipilimumab and nivolumab antibodies	0.02	Only anthracycline induced CMP in the MMR
Hypersensitivity reaction (drugs)	Azithromycin, benzodiazepine, clozapine, cephalosporin, dobutamine, lithium, diuretics, methyldopa, mexiletine, streptomycin, sulfonamides, NSAIDs, tetracycline, tricyclic antidepressants	0.001	Only one patient with lithium intoxication in MMR
Hypersensitivity reactions (venoms)	Bees, wasps, scorpions, snakes, spider	0	–
Radiation injury	–	0.015	History + biopsy + imaging
Metabolic disorder	Diabetic cardiomyopathy	0.02	History + biopsy + imaging in diabetes patients
4. Other DCM patients	–	16.62	–

^a^The MMR included 1098 patients with the diagnosis of suspected myocarditis or inflammatory cardiomyopathy who were examined during 1990–2010 (modified from [1, 2, 4]). Diagnoses were made in most cases via left or right ventricular EMB with PCR, histology, and immunohistology or conclusive serodiagnosis including cardiac autoantibodies

*CMP* cardiomyopathy, *DCM* dilated cardiomyopathy, *Echo* enteric cytopathic human orphan virus, *EMB* endomyocardial biopsy, *ELISA* enzyme-linked immunosorbent assay, *IGRA* interferon-gamma-release assay, *ISH* in situ hybridisation, *NSAIDs* nonsteroidal anti-inflammatory drugs, *PCR* polymerase chain reaction, *pos.* positive

## Special considerations for complex diagnoses

Whether *diabetic cardiomyopathy* is a diagnosis of its own is still under discussion. In endomyocardial biopsies of patients with heart failure and diabetes, histology can show microangiopathy, some infiltrating macrophages and leukocytes, and also a positive PCR of viral genomes such as parvovirus B19. Diabetic cardiomyopathy can be part of a syndrome comprising hypertrophy and microangiopathy due to hypertensive heart disease and diabetes and viral persistence [7]. For diagnosis of the underlying etiology, a composite view of the clinical evidence and exclusion of other causes of cardiomyopathy by endomyocardial biopsy can be an important clue. However, behind the curtain of diabetic cardiomyopathy, viral heart disease with or without inflammation can be hidden. But which of the factors is then the major etiological determinant?

This issue also holds true for *alcoholic cardiomyopathy *[8]. In these patients, alcohol consumption of more than 40 g/day in men and more than 20 g/day in women for more than 5 years is the somewhat arbitrary diagnostic determinant for the label of alcoholic cardiomyopathy. In endomyocardial biopsy, some infiltrating leukocytes may even suggest myocarditis in immunocompetent alcohol-dependent individuals as a likely differential diagnosis.

## Clinical syndromes associated with inflammatory cardiomyopathy and myocarditis

**Fig. 1:**
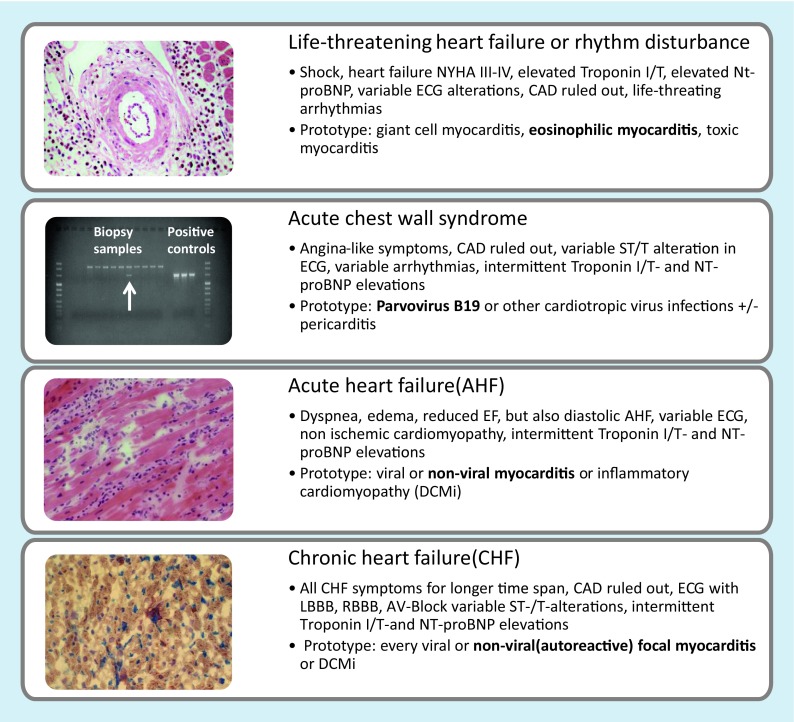
Clinical and histological phenotypes of myocarditis and inflammatory cardiomyopathy. *CAD* coronary artery disease, *ECG* electrocardiogram, *EF* ejection fraction, *LBBB* left bundle branch block, *NYHA* New York Heart Association, *RBBB* right bundle branch block

Depending on the etiology, genetic predisposition, and comorbidities of the individual patient, at least four clinical syndromes can be identified after coronary artery disease is excluded by angiography (Fig. 1):

1. Life-threatening heart failure or rhythm disturbance
2. Acute chest wall syndrome with angina pectoris-like symptoms, often after an infection
3. Acute onset of heart failure
4. Chronic heart failure

Table 2 connects these clinical syndromes with classic textbook diagnoses such as fulminant, acute, chronic, or persistent chronic myocarditis.

**Table 2:** Phenotypes of myocarditis and treatment options (modified from [1])

Clinical phenotype	Fulminant myocarditis	Acute myocarditis	Chronic active or persistent myocarditis
Syndrome	Life-threatening heart failure or rhythm disturbance	Acute chest wall syndrome or acute onset of heart failure; pericardial effusion (up to 10%); angina in parvovirus B19 myocarditis	Chronic heart failure, variable EF with LV dilatation, pericardial effusion (up to 10%); angina in parvovirus B19 myocarditis
Dallas criteria [9]	Infiltrate (active myocarditis or giant cells), necrosis	Active, often focal lymphocytic myocarditis	Borderline myocarditis, focal small infiltrates
World Heart Federation criteria [10, 11]	≥50 infiltrating cells/mm^²^, necrosis, possibly giant cells	≥14 infiltrating cells, mostly lymphocytes, necrosis, necrosis likely	≥14 infiltrating cells, lymphocytes and macrophages, necrosis and apoptosis not obligatory
Immunohistology	Immunoglobulin binding mostly IgM to sarcolemma and fibrils and complement fixation	Immunoglobulin (IgM, IgA and IgG) binding to sarcolemma and fibrils	Immunoglobulin (IgG) binding to sarcolemma and fibrils
PCR of microbial pathogens	Negative in giant cell or autoreactive myocarditis, positive in up to one third of cases	Negative in autoreactive lymphocytic myocarditis, positive in up to one third of cases	Negative in autoreactive lymphocytic myocarditis, positive in up to one third of cases
Course	Variable: from fatal outcome to spontaneous healing	Variable: from deterioration to defective healing	Chronic heart failure
Treatment	1. Immunosuppression in PCR-negative cases, 2. In virus-positive biopsies; ivIg, 3. In all patients: assist device and ICDs, if indicated; heart failure treatment	1. Immunosuppression in PCR-negative cases, 2. In virus-positive biopsies; ivIg, 3. In all patients: assist device and ICDs, if indicated; heart failure treatment	1. Immunosuppression in PCR-negative cases, 2. In viral myocarditis ivIg or IFN in controlled trials 3. In all patients: prophylactic ICDs, when EF < 35%; heart failure treatment

*EF* ejection fraction, *ICDs* implantable cardioverter-defibrillators, *IFN* interferon, *ivIg* intravenous immunoglobulin, *LV* left ventricular, *PCR* polymerase chain reaction

## Treatment

### Restriction of physical activity

In suspected or histologically validated myocarditis, restriction of physical activity for at least 6 months is part of the international guidelines. This is highly recommended until the inflammation has disappeared—evidenced by cardiac MRI or endomyocardial biopsy—and cardiac function has normalized.

### Heart failure therapy for inflammatory cardiomyopathy

Heart failure therapy is part of the treatment of inflammatory cardiomyopathy. It was successfully demonstrated in many heart failure trials on angiotensin-converting enzyme (ACE) inhibition such as the CONSENSUS trial with enalapril, the SOLVD trial with captopril, the ATLAS trial with lisinopril, or the HOPE trial with ramipril. In the CHARM and ELITE II trials, angiotensin receptor blockers demonstrated a similar benefit. Today, beta-blockade is part of the therapeutic armamentarium in the treatment of any form of heart failure as demonstrated in the MERIT-HF trial for metoprolol, the CIBIS trial for bisoprolol, and the COPERNICUS trial for carvedilol. In acute cardiac decompensation, loop diuretics are effective and aldosterone receptor blockers should be given on top of the other heart failure drugs as demonstrated by the RALES trials for spironolactone in heart failure and by the EPHESUS trial for eplerenone in heart failure patients after myocardial infarction. According to the findings of the SHIFT trial, ivabradine can be given to treat sinus tachycardia and to reduce heart rate to below 70 bpm. Cardiac glycosides were tested in the DIG trial, which demonstrated a reduction of all-cause and heart failure-related hospitalization with no change in mortality rate. Their use in patients with tachyarrhythmia reduces heart rate and improves the quality of life.

Antiphlogistic treatment with nonsteroidal anti-inflammatory drugs (NSAIDs) such as ibuprofen or indomethacin should be reserved for patients with pericardial involvement, since in murine coxsackie B3 myocarditis this treatment was shown to be detrimental [12]. For treatment of peri(myo)carditis, we prefer colchicine instead, not only in recurrent forms but also for the first attack [13].

### Antiarrhythmic treatment

Apart from beta-blockers, antiarrhythmic treatments for heart failure and for cardiomyopathy patients have been disappointing. A meta-analysis of all trials with amiodarone demonstrated a reduction in total mortality of 13% [14], but the SCD-HeFT trial, in which patients with a single-chamber implantable cardioverter-defibrillator (ICD) were randomized to amiodarone or to placebo, showed a decrease in mortality for the treatment group only [15]. The discussion of whether rate or rhythm control is more beneficial in the treatment of atrial fibrillation is still ongoing. Sufficient anticoagulation is important under all circumstances.

### Device therapy

In patients with dilated cardiomyopathy with or without inflammation, antibradycardia pacing in second- and third-degree atrioventricular block or in bradyarrhythmia is well established. If the ejection fraction (EF) is below 35% and acute myocarditis is diagnosed, cause-specific treatment should be carried out with a LifeVest wearable defibrillator. If inflammation has disappeared and cardiac function remains low (EF < 35%), the implantation of an ICD is warranted according to current guidelines [16].

## Immunosuppressive treatment

### Idiopathic giant cell myocarditis

If untreated, the natural course of giant cell myocarditis is fatal in almost all cases [17]. The few patients in the MMR were treated with a combination of prednisone and azathioprine (see autoreactive myocarditis). The maintenance doses of prednisone (7.5 mg/day) and azathioprine (50 mg/day) were given as a life-long therapy. All patients received an ICD and have survived 5 years without heart transplantation.

### Cardiac sarcoidosis

In cardiac sarcoidosis the infiltration of cells including giant cells is confined to the noncaseous granuloma. In the MMR, cardiac sarcoidosis was six times more frequent than giant cell myocarditis. The treatment algorithm is either corticoid therapy alone or in combination with other immunosuppressive drugs, e. g., azathioprine or cyclosporine [18].

### Eosinophilic heart disease

Eosinophilic heart disease (EHD) and the resulting endomyocardial fibrosis are rare diseases. Its common pathogenetic denominator is the overproduction of cytotoxic eosinophils [19].

Our experience with long-term prednisone and azathioprine documents a survival rate of 9 out 10 cases over a mean period of 8.4 years [20].

### Treatment in autoreactive, lymphocytic myocarditis

#### Immunosuppression

No randomized or blinded treatment trials have been published in the past 6 years with respect to immunosuppressive therapy in myocarditis.

Viral infection, according to common belief, may trigger an autoreactive cellular and humoral immune response that leads to myocardial damage with inflammation. Following this pathogenetic hypothesis, immunosuppressive treatment either by prednisone alone or in combination with azathioprine or cyclosporine was examined in five trials, the results of which are summarized in Table 3.

**Table 3:** Trials on immunosuppressive treatment

Author	Treatment	Endpoint	Patients/controls (*n*)	Result	Comment
Parillo et al. [21]	P	Function + mortality after 3 months	60/62	Improved 67%	No viral PCR
Mason et al. (MTT) [22]	P + A/CyA	Function, mortality	64/47	No benefit, no harm	Underpowered, no viral PCR
Wojnicz et al. [24]	P +A	EF + function, mortality	41/43	EF improved	No viral PCR
Frustaci et al. (TIMIC) [25]	P +A	EF + mortality after 6 months	43/42	88.3% improved	Treatment in virus-negative pts. only
Maisch et al. (ESETCID) [26]	P +A	EF + function, MACE	54/47	EF + function improved after 2 years	Treatment in virus-negative pts. only

*A* azathioprine, *CyA* cyclosporine, *EF* ejection fraction, *MACE* major adverse cardiac events, *P* prednisone, *PCR* polymerase chain reaction

The first randomized, placebo-controlled trial on prednisone in myocarditis was conducted by Parillo et al. [21], who treated 60 patients with inflammation and 62 without inflammation out of a dilated cardiomyopathy cohort of 122 patients with prednisone: 67% of the patients with inflammation who received prednisone and 28% of inflammation controls experienced an improvement in left ventricular EF of >5% (*p* = 0.004). The Myocarditis Treatment Trial (MTT) by Mason et al. in 1995 [22] showed neither a benefit nor an increased mortality after a 6-month treatment with cyclosporine A or azathioprine and prednisone when compared with placebo. However, the study was underpowered and did not distinguish viral from nonviral disease, as pointed out in a letter to the editor [23]. In the first 6 months of the immunosuppressive therapy, the MTT showed a trend for the benefit of immunosuppression with respect to transplant-free survival, but it missed statistical significance by one patient. In the later follow-up, the results remained neutral.

Wojnicz et al. randomized 84 patients with dilated heart muscle disease and increased human leukocyte antigen (HLA) expression for a treatment of azathioprine and prednisone or placebo for 3 months. In the treatment group, EF improved and survival remained comparable between the placebo and verum group [24].

In the TIMIC study, Frustaci et al. reported that the EF of 43 patients in the treatment group improved from 26.5% at baseline to 45.6% at 6 months (*p* < 0.001). Similarly, left ventricular end-diastolic volume, left ventricular diameter, and New York Heart Association class improved significantly [25].

The ESETCID (*E*uropean *S*tudy on the *E*pidemiology and *T*reatment of *C*ardiac *I*nflammatory *D*isease) is a double-blind, randomized, placebo-controlled three-armed trial with prednisolone and azathioprine for autoreactive (virus negative) inflammatory dilated cardiomyopathy in patients with an EF below 45% at baseline. Interferon alpha is given in enteroviral myocarditis, and intravenous immunoglobulins (ivIg) are given in cytomegalovirus, adenovirus, and parvovirus B19 myocarditis, vs. a placebo drug. The intermediate analysis of the immunosuppressive treatment arm showed a positive trend for EF and major adverse cardiac events after 6 months of treatment and significant benefit after 1 year of follow-up for both groups [26]. Remarkably, the control group also showed also some spontaneous resolution.

#### Intravenous immunoglobulins

ivIg have demonstrated benefit in various inflammatory settings, clinically and experimentally. Treatment with ivIg relies on a polypragmatic therapy approach: IvIg interact widely with the immune system. In addition to immunoglobulin G (ivIgG), the IgGAM Pentaglobin®, in even lower concentrations than ivIgG, exerts proinflammatory and anti-inflammatory effects. This has been shown in sepsis and also in viral heart disease both clinically and experimentally. Proinflammatory effects are the activation of immune cells and of the complement system and the opsonization of infective agents [27]. Anti-inflammatory effects comprise the neutralization of bacterial and other toxins, of degradation products, and of an excess of complement factors and cytokines. This can stimulate immune cells to set anti-inflammatory cytokines such as interleukin (IL)-1RA and IL-8 free and inhibit the liberation of proinflammatory cytokines, e. g., IL-6 and IL-1 [1]. Anthony et al. [28] have shown that the anti-inflammatory activity of monomeric IgG is completely dependent on the sialylation of the *N*-linked glycan of the IgG Fc fragment. The IgM fraction in ivIgGAM can play a distinct role in controlling inflammatory and autoimmune disease. Furthermore, IvIgGAM can reduce oxidative stress [29]. Its effect has been shown in heart failure[30–34], in peripartum cardiomyopathy [35], in fulminant [36–38], acute [30, 39–46], and chronic myocarditis [38], in dilated cardiomyopathy [46], as well as in enteroviral [47] and in parvovirus B19-associated heart disease [48, 49]. IgM-enriched immunoglobulins appear to be effective in lower doses [34], which corresponds to our own observation with Pentaglobin®. Table 4 gives an overview of the ivIg studies. Not all studies reported hemodynamic benefit or improvement, however: The IMAC, a randomized controlled trial, demonstrated improvement in both the treatment and placebo arm [42], so that in a recent multi-institutional analysis [50] the benefit in a pediatric population was questioned.

**Table 4:** Registries and trials with ivIg in inflammatory cardiomyopathy or myocarditis

Authors	Study design	Patients (*n*)	Histology /PCR	ivIg dose	Outcome
Drucker et al. [40]	Retrospective	46 children	Partly, no PCR	2 g/kg single dose	Reduced LVEDD
McNamara et al. [41]	Uncontrolled	10 adults	Partly, no PCR	2 g/kg single dose	Improved EF
McNamara et al. [42]	RCT IMAC	62 DCM, only 13 myocarditis	No PCR	2 g/kg single dose	Both groups improved
Kishimoto et al. [30, 46]	Case series	Total 9, 4 myocarditis	No PCR	1–2 g/kg single dose	Improved NYHA and EF
Dennert et al. [49]	Uncontrolled	25	PVB19 positive	2 g/kg single dose	Decreased viral load, improved EF
Maisch et al. [51]	Uncontrolled	90 PVB19 36 ADV	PCR-positive for PVB19 and ADV	20 g per person at day 1 and 3	Improved EF in 90%, eradication of ADV in 90%, of inflammation in 100%; PVB19 eradication in 40%, of inflammation in 70%
Maisch et al. [52]	Controlled	18/17	CMV by PCR	14 days, multiple doses	Improved EF, complete CMV eradication

*ADV* adenovirus, *CMV* cytomegalovirus, *DCM* dilated cardiomyopathy, *EF* ejection fraction, *ivIg* intravenous immunoglobulins, *LVEDD* left ventricular end-diastolic diameter, *NYHA* New York Heart Association, *PCR* polymerase chain reaction, *PVB19* parvovirus B 19

The MMR data support a positive effect of 20 g i. v. pentaglobin in adenovirus-positive myocarditis for clinical improvement, with eradication of both the inflammation and the virus [51]. In parvovirus B19 myocarditis, our data indicate a clinical improvement; however, only inflammation is successfully eliminated, whereas parvovirus B19 persistence remains a problem in many patients although the viral load is often decreased.

#### High-dose ivIG in cytomegalovirus myocarditis

In biopsy-proven cytomegalovirus (CMV) myocarditis, one controlled trial of 18 patients reported on the eradication of inflammation and elimination of the virus [52]. The patients had received 2 ml/kg i. v. cytomegalovirus hyperimmunoglobulin (CMVhIg) for 3 days and 1 ml/kg for an additional 2 days, alternately.

In parvovirus B19-associated inflammatory dilated cardiomyopathy, dose-finding studies and randomized trials are still lacking and should be planned in the future.

#### Antiviral treatment with interferon beta

In the BICC trial, patients with enterovirus-, adenovirus-, and parvovirus B19-positive genomes received either 4 × 10^6^ or 8 × 10^6^ IU interferon beta-1b vs. placebo [53]. In the small enteroviral and adenoviral myocarditis strata, interferon-beta tended to eliminate the viral genome, to decrease inflammation, and to improve hemodynamics, whereas in parvovirus B19 and human herpesvirus 6 myocarditis, the response was disappointing. For all three viruses, viral elimination or viral load reduction was higher in the interferon beta-1b treatment group than in the placebo group, but least effective in the parvovirus B 19 treatment arm.

## Practical conclusion

- In inflammatory dilated cardiomyopathy and myocarditis, apart from heart failure and antiarrhythmic therapies, there is no real alternative to an etiologically driven specific treatment.
- Diagnosis of the underlying microbial agent is a prerequisite for the initiation of treatment with antiviral agents or ivIg, which is the focus of this review.
- If no virus but autoreactive myocardial inflammation is identified, immunosuppressive treatment is the treatment of choice.
